# Unlocking insights: assessing the quality of conventional and image-based responses on books at home in an online mobile survey

**DOI:** 10.1007/s11135-025-02519-7

**Published:** 2026-01-03

**Authors:** Patricia A. Iglesias

**Affiliations:** 1 Centre d’Estudis Demogràfics (CED-CERCA), Barcelona, Spain; 2 RECSM, Universitat Pompeu Fabra, Barcelona, Spain

**Keywords:** (Visual) data quality, Quality indicators, Image collection, Mobile online surveys, Books at home, Measurement errors

## Abstract

Despite growing interest in collecting photos within online surveys, little is known about the quality of visual data and its comparison with data obtained through conventional requests. To address this gap, a self-administered online mobile survey targeting parents of children attending primary school in Spain was conducted through the Netquest opt-in panel in 2023. The survey gathered information about books in respondents’ homes through photos and conventional questions. First, a review of previous research using conventional questions, photos, and other emerging data types was conducted to identify indicators suitable to evaluate the quality of the information about books at home collected through conventional and image-based formats. Second, most of these indicators to measure quality were estimated. Results reveal important measurement errors in conventional questions, while photos submitted by respondents are generally in line and can be classified. However, concrete information of interest about the books, such as the intended audience or languages, is often difficult to extract from photos. When comparing quality, conventional answers provide more information about the items asked than photos, but photos have the potential to provide additional insights, such as book titles. Overall, while collecting and analyzing photos sent through surveys presents challenges, their integration into surveys offers unique opportunities to enrich data collection methods.

## Introduction

Surveys are widely used across disciplines (Saris & Gallhofer 2014), but they suffer from errors (Groves et al. 2009). The growing use of smartphones can help mitigate these issues by collecting new data types through sensors like microphones or cameras.

Visual data generate particular interest because they might reduce respondent burden, social desirability bias, memory-related errors, and response mistakes. Additionally, they can capture information unknown to respondents (e.g., dangerous moles) and provide new insights (Revilla 2022).

Photos have been used as a source of information in various fields, including social sciences where visual data are often analyzed from qualitative perspectives. To illustrate, visual anthropology uses diverse visual methods, such as digital storytelling and Photovoice, to explore human behavior, experiences, and tacit knowledge (Pink 2011). For example, Chaney and Goulding (2024) have investigated emotions at rituals like rock festivals, where participants photograph and contextualize their experiences. However, some disciplines, such as sociology, remain cautious about visual methods, questioning their validity and representativeness; furthermore, visual data—particularly photographs—often require significantly more inference and interpretation than other types of data (Grady 2008).

In the field of survey research, literature remains sparse. Early research regarding visual data collection through online surveys has mostly examined respondents’ willingness to share images or videos (Revilla et al. 2019; Struminskaya et al. b; Wenz et al. 2019), burden perception, availability and skills to do so (Iglesias & Revilla 2024), and actual participation in questions requesting visual data (Bosch et al. 2019, 2022; Ilic et al. 2022; Ochoa & Revilla 2025; Struminskaya et al. 2021a).

Consequently, little is known about the quality of information gathered in surveys through photos and its comparison to conventional questions. This paper assesses the quality of these methods when asking about a common social sciences topic: the number (as well as languages and storage) of books at home.

The number of books at home is often used as an indicator of cultural and/or economic capital (Heppt et al. 2022; Sieben & Lechner 2019), usually measured in surveys with intervals as response categories. For example, Sieben and Lechner (2019) used 0–10, 11–25, 26–100, 101–200, 201–500, and > 500 books, while Sanders et al. (2004) asked with open-ended questions.

However, respondents are unlikely to know the exact number of books at home and might overreport due to social desirability bias (see Iglesias 2024). Moreover, book quantity might not fully capture cultural capital: details regarding the content are important, but difficult to obtain with conventional questions.

Given the familiarity with capturing and sharing photos via smartphones (Iglesias & Revilla 2024), asking participants to send photos of their books could mitigate these limitations. Photos could provide more precise counts and additional insights, like titles, languages, or storage system.^1^ Yet data quality depends on classification—how information is extracted from photos (Bandyopadhyay 2021)—alongside other factors, like the collection tool and image clarity (Iglesias et al. 2024).

While image quality has been studied in other fields, it has not been evaluated from a survey perspective. However, photos collected through surveys have unique characteristics: they are typically provided by non-expert photographers, so low visual clarity could be common; they may include overlapping items if respondents submit multiple images, which can be problematic when counting items; and they are intended to address research questions distinct from those in fields like computer vision, which often focus on technical analyses rather than population characterization.

In this paper, I conducted a literature review on indicators used to assess the quality of conventional survey questions, photos, and other emerging survey data types. I also explored image-related indicators outside survey methodology. Then, I adapted these indicators to the books case and, when possible, I estimated and contrasted the quality of information from conventional and image-based formats, using data from a self-administered online mobile survey targeting parents of primary school children in Spain.

Results show that both conventional and image-based questions involve important measurement errors, though in different forms. However, photos can reveal extra information, such as book titles.

## Background

Quality has long been a key focus in survey methodology, but it “is a vague, albeit intuitive, concept with many meanings” (Lyberg 2012, p. 107). Accordingly, multiple approaches exist to define and measure it.

### Quality assessment of conventional questions

Two main approaches have been applied to survey data quality: the Total Survey Error (TSE) framework and the use of indicators suggesting potential errors.

#### TSE framework

The TSE framework (Groves et al. 2009) differentiates errors on two sides: measurement and representation. Representation errors include coverage, sampling, nonresponse, and adjustment; measurement errors involve validity, measurement and processing. I mostly focus on the measurement side.

Validity (or “conceptual validity”) refers to “the extent to which the measure is related to the underlying construct” (Groves et al. 2009, p. 50). Assessing the validity of the number of books at home involves determining how well it reflects participants’ cultural and/or socioeconomic capital.

For instance, Siebens and Lechner (2019) investigated the **convergent** and **divergent validity** of the number of books as a measure of cultural capital. The authors examined how the past and present number of books related to variables either associated with cultural capital (convergent validity) or not (divergent validity).

This paper mostly focuses on the second component of the measurement side: measurement errors, defined as the gap between the true value of a measurement and the observed one (Groves et al. 2009). For example, measurement errors occur if the true number of books differs from the reported one. There are two main kinds of measurement errors: random and systematic. Random errors occur when respondents make accidental mistakes (e.g., typing 35 instead of 305 books), while systematic errors reflect consistent reactions to the method (e.g., repeatedly selecting the middle category of a scale). The complement of random errors is defined as reliability, and the complement of systematic errors is termed (measurement) validity (Saris & Andrews 1991). Measurement validity assesses the relationship between a concept of interest (e.g., number of books) and its true score, which is also affected by the method.

**Reliability** was first studied through the test–retest model (attributed to the work of Spearman 1904), which applies the same measure in two moments. Later, more complex models emerged, especially the quasi-simplex model (Heise 1969), which uses at least three measures of the same concept. Researchers have also used the multitrait-multimethod (MTMM) approach (Campbell & Fiske, 1959), which repeats correlated questions using different methods. Reliability and (measurement) **validity** can then be estimated through structural equation modeling (SEM), particularly using the True Score model (Saris & Andrews 1991). The product of reliability and validity represents the overall **(measurement) quality** of a question.

The final component on the measurement side covers processing errors, defined as the difference between the variable used for estimation and the respondent’s answer (Groves et al. 2009). Processing errors can occur when mistakenly correcting cases considered incorrect (e.g., outlier), or when coding answers. For coding tasks, **Interrater Reliability (IRR)** is used to measure coder consistency (McHugh 2012). IRR is usually estimated via percent agreement between coders, Cohen’s kappa (Cohen 1960) for nominal variables, and the Intraclass Correlation Coefficient (ICC; Gisev et al. 2013) for numerical variables.

#### Indicators

While the TSE offers a comprehensive evaluation of survey errors, survey quality is multi-faceted and can be also be examined through indicators assessing how “good” survey answers are.

First, indicators have been used to study the quality of measures based on conventional questions in general:

- **Item nonresponse**: skipping a question without answering (Tourangeau et al. 2018).
- **Non-substantive answers:** responses lacking meaningful content, like “Prefer not to answer” or “Don’t know” (DK) (e.g., Lugtig & Toepoel 2016).
- **Completion time**: measured per question or for the entire survey, as in Wenz (2021), usually assessed using focus time (see Höhne & Schlosser 2017). Answering too quickly may indicate satisficing (Malhotra 2008), while long times might reflect limited skills or comprehension.

High levels of item nonresponse, non-substantive answers, and extreme completion times (too short or long) indicate lower data quality.

Second, some indicators have been used to measure the quality of open-ended narrative questions:

- **In line**: evaluates whether answers align with the question (e.g., Ilic et al. 2022).
- **Answer length:** usually measured by the number of characters/words in an answer (e.g., see Tourangeau et al. 2017).
- **Number of concepts/themes**: since longer answers may arise from repetition, Smyth et al. (2009) proposed counting distinct dimensions covered.
- **Elaboration**: an answer could mention many topics but lack detail. Smyth et al. (2009) also considered whether respondents expand on (adding detail) rather than merely shifting topics.
- **Correctness of spelling, grammar, and punctuation,** as used by Wenz (2021).

Answers in line and with more characters/words, concepts, elaboration, and correctness indicate higher data quality.

Third, other indicators have been used for numeric open questions:

- **Rounding**: to reduce burden, respondents might round answers, often to multiples of five or 10 (Hanisch 2005).
- **Out of range**: numeric values can fall outside the expected range. For example, a number above 10 is out of range for a 0–10 scale (see Couper et al. 2001).

High levels of rounding and out-of-range values indicate lower quality.

Fourth, data quality can also be evaluated across sets of questions, usually with similar response categories, by assessing response styles—consistent tendencies to answer based on factors beyond the question content (Paulhus 1991). Greater response style presence indicates lower data quality. The most commonly studied response styles are:

- **Acquiescence** or “yes-saying”: respondents tend to agree with any statement (Schuman & Presser 1981), especially when agree/disagree or yes/no scales are used.
- The tendency to select the **middle**, **highest,** or **lowest response category** regardless of the item content.
- The tendency to **respond carelessly** (Van Vaerenbergh & Thomas 2013). In general, any type of **non-differentiation** qualifies as a response style (Loosveldt & Beullens 2017). Non-differentiation occurs when respondents presented with a set of questions using similar scales, particularly in grid format, consistently select the same category (pure straightlining) or similar ones (low variance). Examples are found in Tourangeau et al. (2018).

Fifth, **discrepancies in answers** can be assessed when questions are repeated within a survey or when slightly different questions measure the same concept (e.g., see Revilla & Ochoa 2015). Discrepancies indicate lower quality. Further, when different questions measure the same concept, **convergent validity** can be analyzed, meaning that measures of the same construct using different methods correlate (Groves 1989). Higher correlation indicates higher quality.

Finally, some indicators assess the answering process, specifically whether respondents pay attention by assessing their compliance with instructions:

- **Instrumental manipulation check (IMC)**: requires participants to follow instructions (like clicking a button) to confirm they are reading the questions (Oppenheimer et al. 2009). Failing an IMC indicates quality issues.
- **Selecting the required number of responses:** although web surveys can enforce the number of answers for multiple-choice questions, some authors omit this check and instead use compliance with instructions as a quality indicator (e.g., Revilla & Ochoa 2015).

Overall, previous literature has developed many indicators: some estimate the quality of one or a few questions, while others assess respondent engagement with the answering process.

### Quality assessment of images

#### In surveys

There is no quality framework specific to visual data (Daikeler et al. 2024). However, scarce literature on the quality of visual data exists.

Bosch et al. (2019) evaluated whether respondents sent images **in line** with what was requested. Moreover, Bosch et al. (2022) assessed “compliance” (i.e., submitting a photo; thus the complement to **item nonresponse**) and **completion time** of image-based answers.

Similarly, Ilic et al. (2022) assessed **item nonresponse** and whether answers were **in line**. Further, they studied whether photos provided none, partial, or all of the **desired information** on the items of interest, and identified those where **information extraction was impeded** by insufficient visual quality.

Slavec (2024) also studied whether photos were **in line**. Additionally, to confirm **image authenticity, duplicates** were examined with Google Lens to detect internet-sourced photos, and **capture times** verified to ensure photos were taken during the survey.

Moreover, Wenz et al. (2025) compared expenditures reported through photos and manual entries to **benchmark** data, to assess data accuracy. Finally, to check classification consistency, Iglesias et al. (2024) suggested computing **IRR** across coders working on a subsample of photos.

In sum, indicators used to assess the quality of information provided through photos include: in line, item nonresponse, completion time, possibility to obtain the desired information, image authenticity, comparison to benchmark data, and IRR.

#### In other fields

While research on the quality assessment of images collected through surveys is limited, a well-established body of literature exists in other fields, particularly in computer vision. However, no comprehensive framework has been proposed, though relevant criteria can be found. For instance, Golchubian et al. (2021) identified factors that reduce photo quality, such as poor **lighting**, lack of **focus**, suboptimal **formats**, **noise or white grains**, and **motion blur**. Similarly, Tang et al. (2013) evaluated image quality based on aspects such as **composition**, **color arrangement**, and **emphasis on the subject**. These factors can be assessed using algorithms that automatically predict perceptual quality as perceived by humans (Athar & Wang 2019).

Furthermore, Piva (2013) provides an overview of image forensics, a field that examines the history of digital images to verify their **authenticity**. Image forensics examines how images are processed, either to enhance quality or alter content. For instance, double compression can indicate manipulation. While humans can perform some authenticity checks (e.g., detecting light patterns suggesting edits), algorithms are typically implemented for their greater effectiveness (Ferreira et al. 2020). In the context of survey images, image forensics can ensure that photos are unique (not duplicates), unedited, and captured during the survey, echoing Slavec’s approach (2024; see Sect. 2.2.1).

Thus, elements from computer vision and image forensics can help assess image quality in terms of visual aspects and authenticity, both aspects being crucial for minimizing measurement errors.

### Quality assessment of other new data types collected through online surveys

Previous research has assessed the quality of new data types besides visual data. However, studies collecting new data types such as emojis (Bosch & Revilla 2021) or voice (Höhne & Claassen 2024; Revilla et al. 2020) have mainly used indicators already implemented in conventional questions (e.g., item nonresponse or answer elaboration).

An exception is Revilla and Couper’s (2021) study, which proposes assessing potential **problems** when answering through voice, especially technical and context-derived issues (e.g., inability to speak while at work). Even if problems do not directly assess data quality, they help improve understanding of the answering process, context, and differences that might arise across methods.

Overall, the presence of problems when sharing new data types is another indicator that can be useful for photos.

## Research questions

To fulfill this paper’s goals, it is necessary to first identify which indicators allow assessing the quality of the information regarding the books respondents have at home. Thus, my first research question is:

RQ1: Which indicators can be used to assess and/or compare the quality of the books-at-home information collected through conventional and image-based formats?

The suitable indicators can be used to estimate the quality of the information collected through both formats and, where applicable, compare them. Thus, the second research question is:

RQ2: What is the quality of the information about the books at home when collected through conventional and image-based formats, and how do they compare?

Addressing these research questions enriches the limited literature on assessing the quality of information obtained through photos within online surveys. By comparing photos with conventional formats, this study highlights both the contributions and limitations of photos as a new data type. Further, substantive researchers seeking innovative methods to elicit information beyond book counts could enhance measures of cultural or socioeconomic capital by leveraging insights from photos.

## Methods and data

This research is part of a larger study (to consult the full protocol, see Iglesias et al. 2023). This section presents only the aspects relevant to this paper.

### Experimental design

Data on books were collected using conventional and image-based formats, through a self-administered online mobile survey. The conventional format included 11 questions covering:

- *Number of books*: four open-ended numeric questions about 1) total number of books at home, and number of books for 2) toddlers and illiterate children, 3) literate children and teenagers, and 4) general audiences.
- *Language*: three open-ended questions on the percentage of books 1) in Spanish, 2) in one of Spain’ co-official languages (Catalan, Galician, and Basque), and 3) in other languages.
- *Storage*: four radio-button questions on whether books are stored 1) on shelves, 2) inside closets or drawers, 3) on center, coffee, or night tables or over a desk, and 4) elsewhere.

**Fig. 1:**
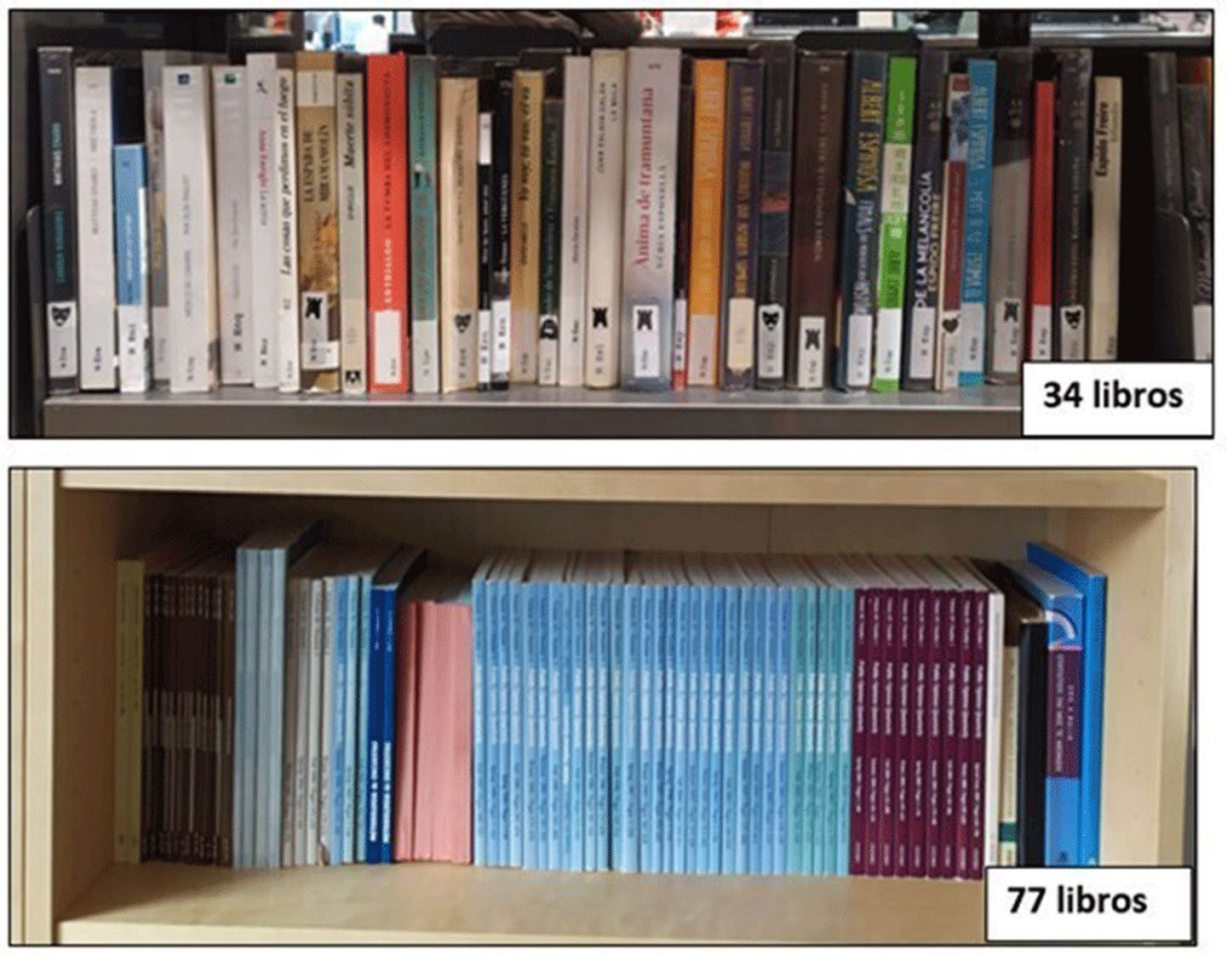
Illustration shown to those answering through *TextPlus*. *Note*: Figure from Iglesias et al. (2023)

For the number of books in the conventional format, two methods were used:

- *Text*: respondents were asked to type the number of books in the four categories.
- *TextPlus:* similar to *Text*, but respondents were presented with an illustration of two equally sized shelves showing different numbers of books (see Fig. 1) to help their estimations.

In contrast, the image-based format used only one method (*Images*), prompting respondents to submit photos of their home books. Detailed instructions and examples of how the photos should or should not look were provided (see SOM1).

Respondents were divided into four groups; this paper focuses on three^2^:

- *Text-TextPlus:* respondents were first asked the conventional questions regarding the number of books, then shown the illustration and asked these questions again, followed by the questions about languages and storage.
- *TextPlus-Images:* respondents were asked all conventional questions (with illustration), followed by the photo request.
- *Images-Text:* participants were first asked to submit photos, and then the conventional questions (without illustration).

### Questionnaire

The questionnaire had up to 65 questions (see SOM2 for original and English translation of the questionnaire). Besides the questions about books at home, it collected sociodemographic variables, characteristics of their child(ren) in primary school, literature-related activities, use and comfort with new technologies, and self-assessments of certain skills.

When starting the survey, respondents encountered a message urging them to respond from home. Nevertheless, since the survey did not collect geolocated data, verifying respondents’ locations was not possible. Consequently, respondents could proceed even if they were not at home.

Additionally, respondents were restricted to complete the survey on mobile devices. Computers were excluded due to the difficulty of using them to photograph home books. Respondents starting on a computer had to switch to a smartphone/tablet to continue. This requirement was not expected to create significant problems since previous research with the same panel showed that most participants used smartphones (e.g., 69% in Iglesias & Revilla 2024). Indeed, 82% of respondents answered the survey without interruptions, indicating that a maximum of 18% began on a computer.

### Data collection

The target population was parents of children in the first, third, and fifth year of primary school in Spain when answering the survey.^3^ Quotas for age, gender, and education were determined using the Economically Active Population Survey by the Statistics Office in Spain,^4^ to reflect characteristics of parents with children aged six to 12 (the typical primary school age range). The survey was conducted in June 2023 using the Netquest opt-in online panel.

Of 4854 invited panelists, 2443 started the survey. 899 were filtered-out due to security checks or eligibility issues (e.g., no child in primary school), 72 because quotas were filled, and 202 abandoned the survey before the first question about books. Therefore, 1270 reached the questions of interest. Since 305 participants were in the group excluded from this paper, 965 individuals are analyzed: 304 in *Text-TextPlus*, 332 in *TextPlus-Images*, and 329 in *Images-Text*. 215 respondents across these groups sent 661 photos; participants in the excluded group sent 63. Thus, 724 images were submitted in total.

Among the 965 analyzed, 54% were female. The mean age was 42, and 44% completed tertiary education. 99% used a smartphone to answer (1% a tablet). For those finishing the survey, the median completion time was 9.6 min.

### Classification

First, the project’s ethics advisor reviewed all photos, blurring personal information (e.g., family portraits) before sharing them with the research team.

Next, two trained researchers manually classified the 724 photos following detailed guidelines (see SOM3 for guidelines and SOM4 for interrater reliability). Each half of the photos was assigned to a classifier, with 100 photos analyzed by both to identify and correct systematic classification differences. Since one group is excluded from this paper, the analysis considers 661 photos, with 92 classified by both researchers. In order to protect respondents’ privacy and following the guidelines established for the project, the photos cannot be shared publicly.

Classification occurred at both photo and respondent levels, accounting for multiple images per respondent to prevent duplicate counting (e.g., overlapping books across different photos).

### Analyses

To answer *RQ1* (indicators of data quality for book questions), a review of books (e.g., Groves et al. 2009; Saris & Gallhofer 2014) and papers on survey data quality, particularly regarding online surveys (e.g., Revilla & Ochoa 2015; Tourangeau et al. 2017) was developed. References within these sources were examined to find new quality indicators. Tools like ChatGPT and Elicit were also used to locate further references on survey data quality potentially missed in prior reviews. Searches with keywords such as “data quality in surveys”, “survey quality,” and “quality indicators” were conducted. This exploration unveiled indicators applicable to the conventional questions about books at home. However, some results were unusable due to unavailability or unverifiable existence, so the core literature remains based on the comprehensive previous review.

For visual data, besides identifying indicators from the conventional question literature, research on quality of visual data within surveys and in other fields was reviewed. Additionally, literature on other new data types, particularly voice, was revised to identify quality indicators adaptable to photos of books.

The most relevant literature to answer *RQ1* was presented in the background section; the full list of the references consulted is in SOM5. The results section explains the selected indicators and their adaptations, where applicable, for the books-at-home case.

To answer *RQ2* (quality of the collected data), the indicators defined for *RQ1* and applicable to the data were calculated. Analyses were conducted using R 4.3.3. The dataset and script are provided in SOM6 and SOM7, respectively.

## Results

### Defining indicators to measure data quality

To answer *RQ1*, Table 1 presents a set of indicators that can be used to measure the quality of the information about books at home collected through conventional and/or image-based formats.

**Table 1:** Indicators to measure the quality of the information on the books at home

Format	Indicator	Implementation
Conventional	Non-substantive answers	% DK
	Rounding	% answers finishing in 0 or 5
	Out of range	% language’s questions > 100
		% sum 3 categories ≠ 100
		Median and mean difference
	Discrepancy of answers	% total no. books reported ≠ sum 3 categories
		Median and mean difference (total no. books reported–sum 3 categories)
		% no. books *Text* ≠ no. books *TextPlus*
		Median and mean difference (no. books *Text* – no. books *TextPlus*)
Image-based	Potential for classification	% photos enough visual quality
		% photos with classifiable information for all items
	In line	% photos including at least one book
	Problems	% technical problems % problems to understand the functioning % unable to photograph all books
		% with at least one problem
	IRR	% agreement ICC or Cohen’s Kappa
	Aspects affecting visual quality	Not estimated
	Image authenticity	
	Comparison to benchmark data	
Both	Item nonresponse	Absence of item nonresponse
	Discrepancy answers	% no. books from images ≠ from *Text(Plus)*
		Median and mean difference no. books from *Images* & *Text(Plus)*
		% languages from images ≠ from *Text(Plus)*
		% storage systems from images ≠ from *Text(Plus)*
	Convergent validity	Not estimated
	Divergent validity	
	Measurement validity and reliability	
	Completion time	

#### Conventional format only

Some indicators evaluate information from conventional questions (*Text* and *TextPlus*). First, **non-substantive answers** can be studied. A DK option was proposed for the four book-number questions (see SOM1 for screenshots). Respondents answering DK were asked for an approximate number in follow-up questions. The categories previously marked as DK appeared with a textbox and a radio button labeled “I cannot give an approximate number either,” also considered as DK.

Thus, two indicators can be computed: the **proportion answering DK for the four-initial book-number questions,** and **for both initial and follow-up questions**. Both are estimated over all respondents answering the first questions and not breaking off in any of them (although they include those with item nonresponse in the follow-up question).

Second, since several questions are numeric, indicators specific to this answer type can be calculated. **Rounding** can be observed in the four book-number questions and the three language questions by estimating the proportion of answers finishing in 0 or 5 over all the answers provided per question, excluding the numbers 0 in all questions and 100 in the questions on proportions. **Out-of-range values** could be assessed in the language questions: as proportions are asked, no category should exceed 100. Thus, the **proportion of answers with values over 100** represent out-of-range answers. Further, since the three categories are exhaustive and exclusive, they should sum to 100. Thus, any **sum of answers different from 100** is considered out of range. Moreover, for out-of-range sums, **the median and mean differences from the expected value (100)** can be calculated**.**

Third, the reported total number of books should equal the sum of the answers to the three questions about different categories. To assess **discrepancies in the total number of books (reported *vs.* sum),** I compute the proportions of respondents whose reported total differs from the sum of the other three answers, among those answering all book-count questions. For those presenting discrepancies, I estimate the differences between reported totals and sums for each respondent, and then compute the **median and mean** of these **differences**.

Further, for the *Text*-*TextPlus* group, the four book-count questions were asked twice (without and with illustration), allowing to estimate the proportion of respondents with **discrepancies for the numbers reported for these four questions between the first (*Text*) and second answer (*TextPlus)***. Among those with discrepancies, the size of the difference is computed per respondent. Then, the **median and mean differences are reported**.

#### Image-based format only

First, each photo’s **potential for classification** can be evaluated, i.e., whether it has enough visual clarity for analysis (complement to Ilic et al. 2022). Photos lacking clarity are excluded from further analysis. Then, for photos with enough visual quality, the **potential for classification of the information of interest,** i.e., whether the photos allow classifying all numbers of books, presence of language categories, and storage systems, can be assessed. Following Ilic et al. (2022), different levels are used: partial (e.g., in one photo some books’ languages were identifiable but others not) and total (e.g., all books’ languages could be identified).

Second, the proportion of photos **in line** (i.e., containing at least one book) can be computed among those with enough visual clarity.

Third, as 92 photos were allocated to two classifiers, IRR can be computed for the 17 classified items (see SOM4), using the percentage of agreement and ICC for numeric variables and Cohen’s kappa for categorical variables.

Fourth, the **presence of problems when submitting photos** can be assessed. Respondents sending at least one photo were asked whether a) their device had technical problems (e.g., malfunctioning camera), b) they struggled to understand how to capture photos, and c) contextual reasons prevented photographing all books (e.g., children sleeping in the room where books are). I compute the proportions of respondents reporting each problem and at least one problem.

Finally, **aspects affecting visual quality (e.g., lighting or blurriness),** and **emphasis on the subject** could have been applied to the books case but were not estimated, because assessing all these different indicators separately was not necessary. What was relevant was to determine whether classification of the information of interest was possible. Thus, a general photo evaluation was conducted to determine the **potential for classification**, assessing the overall visual quality without breaking it into components like lighting or blurriness. Additionally, subject emphasis was measured not by how well the photo highlighted the items of interest, but by whether it contained books (**in line** indicator).

Regarding **image authenticity**, although it could be assessed for this case, it was not examined as unauthentic photos were not expected. Indeed, the tool employed to gather photos within the survey, WebdataVisual (Revilla et al. 2022), automatically opens the camera, making it unlikely that respondents upload external images. While possible, this was not a major concern. Moreover, since assessing **image authenticity** with image forensics shares the goal of detecting **duplicates** and **capture time** (used by Slavec 2024), these indicators are grouped in the same category in Table 1.

Finally, **comparison to benchmark data** was not conducted as the respondents’ actual number of books was not available.

#### Both formats

Some indicators can assess the quality of the information about books at home captured through conventional and image-based formats, enabling comparisons. These indicators are calculated for respondents offered both formats (*TextPlus-Images* and *Images-Text* groups) and not breaking off during the book questions.

The first indicator is **item nonresponse**. To enhance comparability between formats, I estimate the complement of item nonresponse: the proportion of respondents with information on all 11 items. For conventional questions, to qualify as complete, respondents had to a) provide substantive answers to the four book-count questions (DK excluded), b) answer the three language questions or provided responses summing to 100 (e.g., 100% Spanish implies zero for the other language questions), and c) answer the four storage questions. For photos, completeness required that a) all books could be counted and categorized, and the presence or absence of all b) languages and c) storage systems could be identified.^5^

Further, the proportion of respondents with **discrepancies in the number of books (total and by category)** between photos^6^ and conventional answers can be estimated. For discrepant cases, the **difference in the number of books between methods** is computed per respondent. The within-subject mean and median of these differences are reported. **Discrepancies in the presence of books in each language category** and **storage** can also be studied.^7^ The percentages of respondents with discrepancies are calculated separately by group, as order effects may arise since some started with the conventional questions and others with the photo question. Differences in the levels of discrepancy between groups are tested with Chi-squared tests (5% significance level).

Moreover, **convergent validity** could be studied by analyzing the correlations between methods (*Text, TextPlus,* and *Images*) when measuring the concepts of interest (e.g., numbers of books). **Convergent** and **divergent validity** could also be assessed by examining the correlations between the total number of books measured through the three methods and variables that are theoretically expected to be either related or unrelated (see Siebens & Lechner, 2019). However, these analyses will be conducted in separate papers with a substantive focus. As this paper is the first attempt to systematically identify quality indicators for photos as a new data type, the focus here is on assessing quality without evaluating their interaction with other questions that should or should not correlate with the number of books. Although such an analysis is interesting, it would require a different theoretical background, which is beyond this paper’s scope.

**Measurement validity** and **reliability** could be estimated with SEM since an MTMM design was used, repeating correlated traits with different methods. However, MTMM analyses were unsuccessful, particularly due to the low number of observations in some split-ballot groups. Reliability could also be estimated with test–retest or quasi-simplex models, but the survey design did not allow for such analyses.

**Completion time** may also indicate quality: rapid responses might suggest that participants did not capture photos of all books or did not give sufficient consideration to conventional questions. However, this analysis was not implemented due to paradata limitations.

Finally, other indicators reviewed in the background are excluded in Table 1 as they were not applicable to the books-at-home case (see SOM8).

### Measuring quality of the information about books at home

To answer *RQ2*, I measure the quality of the information about books using the previously defined indicators and compare formats where possible.

#### Conventional answers

Table 2 presents the proportions of **non-substantive** (DK) and **rounded answers**, whereas Table 3 presents those for **out of range** and **discrepancies**.

**Table 2:** Percentage of DK and rounded answers (number of observations in parentheses)

Variable	% DK initial		% DK two questions		% rounding	
	Text	TextPlus	Text	TextPlus	Text	TextPlus
# total books	36	25	13	12	75	77
	(601)	(633)	(601)	(630)	(602)	(630)
# books illiterate children	29	20	13	11	43	54
	(601)	(633)	(600)	(631)	(601)	(631)
# books literate children/teenagers	31	20	14	11	65	71
	(600)	(633)	(600)	(629)	(600)	(629)
# books general audience	42	26	19	15	59	66
	(600)	(633)	(600)	(631)	(600)	(631)
% Spanish					47 (931)	
% co-official languages					29 (700)	
% other languages					40 (754)

**Table 3:** Out of range and discrepancies between answers in the conventional method

Indicators	Estimations	n	%	Median dif	Mean dif
Out of range	Language’s questions > 100	931	0		
	Sum 3 categories ≠ 100	931	6	50	50
Discrepancy	Total # books reported ≠ sum 3 categories (*Text*)	476	57	− 4	− 30
	Total # books reported ≠ sum 3 categories (*TextPlus*)	526	52	− 1	− 17
	Total # books *Text* ≠ *TextPlus*	302	43	− 20	− 28
	# books illiterate children *Text* ≠ *TextPlus*	303	31	− 3	− 2
	# books literate children/teenagers *Text* ≠ *TextPlus*	302	44	10	21
	# books general audience *Text* ≠ *TextPlus*	303	46	13	21

In the initial questions about the numbers of books, **DK** ranges from 20–42%. However, it seems likely that respondents do not know the exact number of books at home. When asked for an approximate number, 11% to 19% of respondents stated DK again.

**Rounding** is frequent: 43–77% of book-count answers are rounded, even among *TextPlus* respondents, despite the illustration showing non-rounded examples. This suggests that most respondents likely provided estimates rather than exact counts. Rounding is lower in the language questions (29–47%), possibly because most books were in Spanish (> 95%).

For **out-of-range** values, no respondent stated a proportion over 100 in the language questions, which is an indication of quality: these questions appeared directly after the number-of-books questions, thus respondents not carefully reading could have answered the book-count in each language instead of the proportion, leading to responses exceeding 100. Furthermore, only 6% of responses do not sum to 100. However, among these, mean and median differences from 100 are both 50 percentage points. While few respondents provided out-of-range values, those who did gave answers far from the expected range.

Regarding **discrepancies** between the stated number of books per category and their sum, important differences emerge (with and without illustration). 52–57% of sums do not match the reported totals. However, median differences for those with discrepancies are small (− 1 in *TextPlus*, − 4 in *Text*) books. In contrast, mean differences are − 30 and − 17 books, respectively. This suggests that while most respondents did not provide accurate numbers for the overall count or category allocation, the size of discrepancies is often reduced.

In the *Text-TextPlus* group, where book counts were provided twice (before and after the illustration), 31–46% of respondents gave different answers. Median differences span from − 20 to + 13 books, and mean differences from − 28 to + 21. These variations suggest that while the illustration may have helped improve accuracy, a substantial portion did not provide precise numbers in the initial question.

#### Image-based answers

Regarding the **potential of classification**, Table 4 shows that 99% of photos have enough visual quality for analysis. Of them, 99% are **in line** with the request.

**Table 4:** Quality indicators for image-based answers

Indicator	n	%
Enough visual quality	661	99
In line	657	99

Potential of classification	n	% total	% partial
# of books	648	69	31
Categorization of books		64	36
Languages		39	59
Storage		95	3

Respondents facing…	n	%
Technical problems	211	5
Understanding problems		4
Unable to capture all photos		21
At least one issue		27

IRR among 17 aspects coded	n	% agreement	ICC/ Kappa
Minimum	90–92	23	0.0
Maximum		100	1
Median		83	0.5
Mean		74	0.4

For photos of enough visual quality and in line, the **potential for classification** of the items of interest was studied. The classification of storage presented the fewest obstacles, with 95% of photos allowing total classification. In contrast, the total number of books was fully classified in 69% of photos and partially in 31%, often limited by objects covering the books (e.g., portraits). Further, all books could be categorized in 64% of photos, while in 36% only some books could. Furthermore, 39% of the photos enabled complete identification of languages, and 59% partial identification, meaning 98% of photos revealed at least some titles. This is important since collecting titles via conventional questions would be too burdensome.

Regarding **problems**, 27% of respondents reported facing at least one. 21% could not photograph all their books due to contextual constraints, 5% had technical issues, and 4% reported understanding difficulties.

As for **IRR,** SOM4 presents the percentage agreement and Cohen’s Kappa/ICC for the 17 variables classified. In average, the agreement between classifiers is 74%, and the Cohen’s Kappa/ICC 0.4, indicating low to moderate consistency between classifiers. Some variables, particularly the book categorization, presented challenges (23–60% agreement; − 0.1 to 0.06 ICC). Overall, classification proves to be difficult, which could impact subsequent analyses.

#### Both formats

First, Table 5 presents **item nonresponse** results. In the conventional format, 76% of respondents provided information for the 11 items of interest compared to 4% for photos (11% if the estimation is only over those submitting photos, n = 214). The latter proportion is low because: a) only 36% of respondents sent photos (for details on participation, see Iglesias 2024), b) many books were unclassifiable, and c) the presence or absence of certain languages could not be identified.

**Table 5:** Proportion of respondents with data on the 11 items

	Conventional	Image-based
All information (%) *(n* = *602)*	76	4

Second, Table 6 presents the results for **discrepancies**. For instance, in the *TextPlus-Images* group*,* the median number of total books is 100 in the conventional format and 96 in photos. For 99% of respondents, the total number of books differs between methods. For discrepant cases, when estimating the size of the differences between the numbers of books obtained through each format per person, median and mean differences are 14 and 47, respectively.

**Table 6:** Discrepancy of answers for conventional and image-based formats

	*TextPlus-Images*						*Images-Text*					
Variable	*n*	*TextPlus*	*Images*	Discrepancy (%)	Median dif	Mean dif	*n*	*Images*	*Text*	Discrepancy (%)	Median dif	Mean dif
Total # of books	88	100	96	99	14	47	103	75	100	100	9	22
For illiterate children	90	10	3	87	2	− 7	102	4	8	82	3	1
For children/teenagers	90	30	15	99	14	10	100	14	30	98	13	14
For general audience	84	45	26	99	15	63	95	20	30	98	10	35
Spanish (%)	84	100	99	1			105	98	100	2		
Other co-official language (%)	84	32	19	73			106	25	38	69		
Other language (%)	84	58	52	57			106	40	55	63		
Shelves (%)	92	97	97	**4**			110	91	87	15		
Tables (%)	92	58	18	46			110	12	57	52		
Closets (%)	92	54	3	53			110	7	56	55		
Other places (%)	91	17	7	25			110	5	16	24		

The columns *TextPlus, Images,* and *Text* express the method used to obtain information about books and present the median number of books, and proportions of languages and storage system. Bold represents statistically significant differences between proportions of Discrepancy (Chi-squared test, 5% level)

Between 98–100% of the reported book counts differ from those extracted from photos, except for illiterate children’s books, where this discrepancy is slightly lower (82–87%). Discrepancies are not significantly different between groups. The median difference for the number of books in the different categories varies from 2–15. For the total number of books, the median difference is 9 in *Images-Text* and 14 in *TextPlus-Images*. These differences could matter since the median total number of books reported by both groups ranges from 70–92, depending on the method. Greater variation is observed in mean differences (up to 63 books), in a context where 114–162 books are reported on average. Overall, both groups reported more books in the conventional format than with images, suggesting a social desirability effect and/or incomplete photo coverage of books. Differences are greater when respondents started with conventional questions.

In language questions, discrepancies are lower but still considerable in some cases with up to 73% of participants having a language identified in one format but not the other. For Spanish, it was only 2% in both groups.

For storage, shelves and “other places” show the lowest discrepancies (4–25%). Still, the latter doubles in *TextPlus* and triples in *Text*, respectively, compared to photos. Tables and closets appear underrepresented in photos, possibly due to classification difficulties: if respondents opened closets to photograph books, they might resemble shelves. Alternatively, it could indicate that respondents did not capture all books in photos or overstated ownership of books in the conventional format (possibly due to social desirability).

## Conclusions

### Main results

Survey data quality is crucial, yet little research has assessed the quality of visual data compared to conventional questions. This paper first identifies indicators to measure the quality of information about books at home, when provided with a self-administered online mobile survey including conventional and image-based questions (*RQ1*). 18 indicators are proposed:

- Four for conventional questions: non-substantive answers, rounding, out-of-range answers, and discrepancies in responses.
- Seven for photos: potential for classification, in line, problems faced, IRR, aspects affecting visual quality, image authenticity, and comparison to benchmark data.
- Seven for both formats: item nonresponse, discrepancies of answers, convergent validity, divergent validity, measurement validity and reliability, and completion time.

When possible, these indicators were tailored to book questions in this study, yielding 27 specific indicators.

By calculating the suitable indicators, *RQ2* could be answered. For conventional questions, out-of-range answers were minimal, but DK, rounding, and discrepancies were high, especially in matching the sum of numbers of books in the three categories with the stated total.

Almost all photos had potential for classification. Compared to previous studies (Bosch et al. 2019; Ilic et al. 2022; Slavec 2024), higher levels of in-line photos were found (99%). Still, 27% of respondents reported problems when capturing and submitting photos, suggesting an underestimation of books through photos. Moreover, classification yielded positive results for generic dimensions but revealed differences among classifiers for variables like book categorization.

Comparing both formats, only 4% of respondents asked for photos have information on all 11 items, versus 76% in the conventional format. This finding contrasts with the 19% of respondents with full information reported by Ilic et al. (2022). However, Ilic et al. focused on heating systems brands and models rather than 11 items of interest. If only the number and categories of books were required, the percentage would increase to 14%, with counts of books available for 44% of those asked for photos. Moreover, classifying book information is more difficult: there are more books than heating system brands/models and requires aspects such as titles to be visible.

Additionally, book-count discrepancies were common, with mean differences up to 63 books. Overestimation of books is greater when reporting through the conventional format.

Finally, discrepancies were minimal for Spanish but higher in the other languages. For storage, most discrepancies involved tables and closets.

### Discussion

Neither conventional nor photo formats are without errors. When book information was provided through both methods, discrepancies appeared: a mean difference of 63 books between methods could shift estimates by up to two categories within the closed intervals previously used in the literature (see Introduction), which could affect substantive conclusions, even more if these differences are systematic. Additionally, an overestimation of books is present among respondents when answering with conventional questions compared to photos. Then, photos offer a more conservative yet reliable baseline for book numbers, since they record at minimum the volumes shown and are less affected by social desirability bias.

Building on previous literature, these findings offer new insights. While respondents sent in-line photos at higher rates than in prior studies, the completeness of information decreases as more detailed classifications are expected. This suggests images are preferable when seeking accurate yet easy-to-collect and -classify data, or when researchers want additional information unavailable through conventional questions, without losing core data.

However, the findings regarding books at home may not be generalized to other concepts or samples. Previous research has used both opt-in (Bosch et al. 2022) and probability-based (Ilic et al. 2022) panels, which could partly explain the mixed results observed. Yet, no direct comparison of these types of panels regarding visual data quality exists. Even so, the proposed quality indicators provide a valuable framework for future studies across varying topics.

### Practical implications

Since errors occur in conventional and image-based formats, researchers should strive to complement both formats. Wenz et al. (2025) showed that photos plus direct entries yielded higher quality insights than photos alone. Further, to make the best of visual data, researchers might consider extracting additional information beyond what can be asked with conventional questions (e.g., book titles).

However, the benefits and drawbacks of collecting photos should be weighed. While improving accuracy and obtaining higher-quality data are important, respondents’ needs and preferences must be considered (see Iglesias 2024). Balancing representation and measurement when collecting new data types is essential (Couper 2024): pursuing more/better data may exclude some respondents, while maximizing participation might compromise data quality. Therefore, determining for whom and under which circumstances photos are more advantageous than conventional questions should be the focus.

The results of this study can extend beyond visual data: researchers working with emerging data types, like voice or video, can adapt the proposed indicators to assess their data quality. To enable comparisons, I encourage researchers to collect these newer data formats alongside conventional types. This combined approach would help validate the answers and estimate diverse quality indicators, enabling assessment of when conventional or innovative formats are suitable.

Finally, when assessing the collection of photos in substantive research, it is important to focus not only on technological advances but also on how concepts are operationalized. In our study, the number of books matters because it has been used to characterize cultural capital.

### Limitations and further research

Some limitations are shared with those presented in Iglesias (2024), as both studies use the same dataset: use of an opt-in panel, focus on Spain and on parents of primary school children. Future research should test robustness with different samples (e.g., probability-based panels and availability sampling), target populations, and countries.

Other limitations are specific to this study. First, the small number of photos limited the development of more complex quality analysis (e.g., MTMM). Second, photo classification was complex (especially for languages other than Spanish and the book categorization), and despite several revisions, issues persisted. These challenges affected item nonresponse results: if even one book in a photo was uncategorized, the photo’s content was deemed incomplete. Lastly, as technology evolves, results may change due to impacts on photo capture, submission, and classification of photos. Researchers should interpret findings with this in mind and regularly assess and adapt survey photo-capture methods.

Future research could explore alternative classification methods (e.g., machine learning) to address these issues. Automatic classification could also help gain additional insights, like book titles, facilitating the identification of book types, thereby enriching the measure of cultural capital. With the ongoing advancements in Artificial Intelligence (AI), automatic photo classification is likely to become faster and more accurate.

The quality indicators could also be examined against respondents’ sociodemographic characteristics, abilities, and technology use to determine whether certain groups differ in data quality. Further, substantive research is needed to assess the quality of both formats for book counts. In this regard, Volodina et al. (2024) have studied both measures in relation to other survey questions concerning home literacy environment and children’s school grades.

Moreover, further research on how to assess image quality should address the use of AI by respondents. While image forensics and image paradata (e.g., capture time) could help verify photo authenticity, artificial intelligence might challenge these methods by potentially evading detection. This issue is key for future research collecting photos to ensure data integrity and validity.

## Data Availability

All the SOMs, including the dataset, are available at the following repository: https://osf.io/7s5mf/overview.

## Footnotes

1 Obtaining the exact number of books could also enable future evaluation of how using exact book counts rather than intervals might influence research outcomes. However, that goal is beyond the scope of this paper.

2 The fourth group, where participants could choose their preferred method, was used to measure preferences and is excluded here due to small sample size and inability to compare across methods.

3 This target population was selected because of the substantive analyses planned using the same dataset (Volodina et al. 2024).

4 https://www.ine.es/dyngs/INEbase/es/operacion.htm?c=Estadistica_C&cid=1254736176918&menu=resultados&idp=1254735976595#!tabs-1254736030639.

5 Information about respondents sending photos, regardless of whether they included all the information of interest, is provided in Iglesias (2024).

6 Unlike photo-level indicators, these are calculated at the respondent level by aggregating data from multiple photos when respondents sent several.

7 The conventional format captured book distribution across the three language categories, while photo classification only recorded presence of books in each category. Values in photos are yes/no/not sure. Values > 0 in the conventional format were recoded as “yes” (presence) and 0 as “no”. Since these variables are dichotomous, within-respondent median and mean differences were not computed.
